# Video Scene Detection Using Transformer Encoding Linker Network (TELNet)

**DOI:** 10.3390/s23167050

**Published:** 2023-08-09

**Authors:** Shu-Ming Tseng, Zhi-Ting Yeh, Chia-Yang Wu, Jia-Bin Chang, Mehdi Norouzi

**Affiliations:** 1 Department of Electronic Engineering, National Taipei University of Technology, Taipei 106335, Taiwan; 2 College of Engineering and Applied Science, University of Cincinnati, Cincinnati, OH 45219, USA; yehzg@mail.uc.edu (Z.-T.Y.); norouzmi@ucmail.uc.edu (M.N.)

**Keywords:** video scene detection, video temporal segmentation, video structure analysis, video chaptering, video summarization

## Abstract

This paper introduces a transformer encoding linker network (TELNet) for automatically identifying scene boundaries in videos without prior knowledge of their structure. Videos consist of sequences of semantically related shots or chapters, and recognizing scene boundaries is crucial for various video processing tasks, including video summarization. TELNet utilizes a rolling window to scan through video shots, encoding their features extracted from a fine-tuned 3D CNN model (transformer encoder). By establishing links between video shots based on these encoded features (linker), TELNet efficiently identifies scene boundaries where consecutive shots lack links. TELNet was trained on multiple video scene detection datasets and demonstrated results comparable to other state-of-the-art models in standard settings. Notably, in cross-dataset evaluations, TELNet demonstrated significantly improved results (F-score). Furthermore, TELNet’s computational complexity grows linearly with the number of shots, making it highly efficient in processing long videos.

## 1. Introduction

Video scene boundary detection or video chaptering is a fundamental task in video structure analysis that facilitates extracting information from videos and enhances the user experience while browsing videos [1,2]. It has shown that effective video temporal segmentation is as essential as predicting frame-level importance scores in generating video summaries [3].

Videos can be divided into shots, which are uninterrupted sequences of visually similar consecutive frames captured by a single camera. However, when it comes to semantically meaningful storylines, scenes, or chapters that are valuable for information retrieval or video summarization, they often comprise a collection of consecutive video shots that are visually dissimilar. These shots may be recorded from different cameras or various angles, forming a cohesive narrative or thematic unit within the video content [4].

Various researchers have studied shot detection algorithms and established methods for identifying shot boundaries using a variety of features [5,6,7]. Given the similarity of frames within each shot, studying shallow visual features such as color histograms or tracking changes in mutual information of consecutive frames can deliver results on par with similar studies of deep features extracted from pre-trained object classification or action recognition models [8].

In contrast to shot detection, understanding videos at a higher level and distinguishing video scenes as a combination of semantically related shots poses a significant challenge. This task necessitates the integration of diverse information modalities through feature fusion [9]. Therefore, the majority of researchers have attempted to select a subset of features (such as texture, objects, motion, text, or voice) and learn the association between these features based on specific video content categories (e.g., surveillance videos, human activity [10]). Despite extensive research in this field, a standardized framework for high-level temporal segmentation of generic video content, particularly scene boundary detection, is still lacking. Moreover, the existing state-of-the-art models rely on knowing the exact number of scenes in a video to detect video scene boundaries effectively. Unfortunately, this information is not always available and cannot be accurately estimated in many cases.

**Figure 1:**
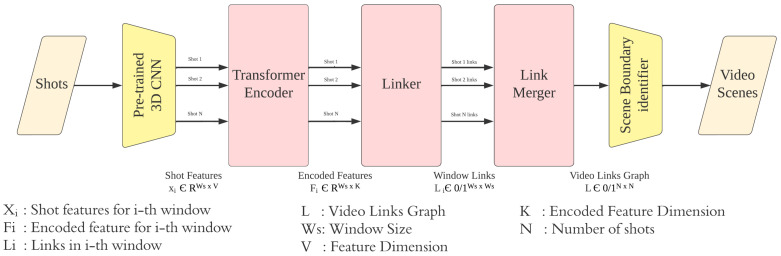
TELNet overall architecture.

Having video shots and focusing on visual features, we propose a transformer encoding linker network (TELNet) that learns associations among shot representations, establishes links among correlated shots, and identifies scene boundaries based on the established links without knowing the number of scenes in a video (see Figure 1). The idea is to have a model which makes intra-scene shot features and shot features that belong to different scenes more distinguishable through feature encoding and generate a graph of shot links for scene boundary identification.

TELNet relies on a pre-trained and fine-tuned 3D CNN model for extracting video shot features and a stack of multi-head self-attention networks for learning associations among the extracted shot features—transformer encoder. The linker network establishes links among shots based on the encoded shot features and creates a graph of shots (nodes) and their edges (links). Scene boundaries are declared where no overhead links exist between consecutive shots. Assuming that each scene can be represented by a key-shot which is the shot closest to the mean of shot features within each scene, video graphs are created and used as a label for the linker network. TELNet scans the video shots in batches (within a rolling window) and aggregates results when reaching the end of the video.

TELNet was trained and evaluated on multiple publicly available datasets and compared to other state-of-the-art models [9,11,12,13,14,15]. TELNet achieved results comparable to the other SOTA models without knowing the number of video scenes in the canonical settings and improved on their results (F-Score) significantly in the transfer setting (cross-dataset evaluations). To summarize, our main contributions are as follows:

- We proposed a transformer encoding linker network (TELNet) that models video shot correlations and identifies video scene boundaries without prior knowledge of video structure, such as the number of video scenes. TELNet’s results demonstrate an increase of 50% in the F-score in half of the transfer settings while maintaining a performance on par with the other SOTA models in the rest of the evaluation settings.
- The transformer encoder and linker were trained jointly in contrast to the other SOTA models, which were trained in increments. TELNet was trained using novel generated graphs in which nodes are the shot representations, and edges are the links among key-shot and other shots within a scene.
- Given that TELNet scans video shots in batches and aggregates the results, the model’s computational complexity grows linearly as the number of shots increases, in contrast to other models whose complexity grows linearly to the square of the number of shots [14], or the NP-hard complexity of the Ncut algorithm that estimates the number of scenes in a video [15]. The prior works of the video scene detection models are shown in Table 1.

## 2. Related Work

### 2.1. Shot Detection

A video shot is the basic video unit comprising a sequence of visually similar frames taken from a single camera, usually at a fixed angle. Given the similarity of frames within a shot, multiple established shot detection algorithms have been proposed for video processing tasks [5]. Chu et al. calculated the pixel-wise RGB difference between consecutive frames [22]. Zawbaa et al. studied the percentage difference of 32 × 32 blocks in HSI space [23]. Potapov et al. proposed a kernel temporal segmentation (KTS) algorithm, which detects shot boundaries distinguishing between step changes due to noise and step changes due to the variation in the underlying multidimensional features [24]. Sokeh et al. determined shot boundaries by applying k-means clustering on frame flow histograms [25]. Even though there are subtle differences among shot detection algorithms, shot boundaries that are identified based on shallow visual features such as color histograms are comparable with shot boundaries identified based on deep visual features [8].

### 2.2. Shot Representation

A video shot can be represented either by a set of frames or a single frame, known as the key-frame. The key-frame, as defined by Rui et al., captures the shot’s salient information, making it a representative sample of the entire shot [4]. Convolutional neural networks (CNNs) designed for image classification have been pre-trained on large-scale datasets, enabling them to learn hierarchical and abstract features from images. Leveraging these pre-trained CNN models allows us to extract deep features from key-frames of video shots, capturing complex visual patterns and semantic information [26]. Baraldi et al. extracted shot visual features using a 2D CNN model trained on ImageNet [27] and the Places datasets [13,28]. Similarly, Protasov et al. extracted frame visual features from a model trained on the Place205 dataset [17]. In another study, Rotman et al. employed Inception-v3 to extract frame visual features [14,20].

It has been shown that 3D convolutional neural networks (3D CNNs) can effectively represent transitions in a sequence of consecutive frames [29,30]. As a result, 3D CNNs have shown impressive performance in action recognition tasks, particularly by tracking objects that appear in the initial frames of a sequence [31,32]. By harnessing the power of pre-trained 3D CNNs, we can extract features representing transitions within shots, offering an alternative or complementary approach to the conventional method of extracting key-frames and using pre-trained image classification models for representation. For instance, Liu et al. fine-tuned a 3D CNN model for representing video shots in their proposed model for detecting movie scenes [15].

Recent studies on shot representation focus on self-supervised representation learning, aiming to enhance network robustness against various variations by modeling representation consistency. Chen et al. introduced a shot encoder incorporating video and audio features [33]. Their approach employs a contrastive learning strategy to identify the most similar shot to the query as its positive key. Subsequently, they maximize the similarity between the query and the positive key while minimizing the query’s similarity with a set of randomly selected shots. For the positive key selection, Wu et al. suggested the scene consistency selection approach [34], which enables the selection to accomplish a more challenging goal. They create a soft positive sample using query-specific individual information and an online clustering of samples in a batch to produce a positive sample.

### 2.3. Scene Boundary Detection

Finding similarities among shot representations can help us identify video scene boundaries [35]. Chasanis et al. used k-means clustering to group the key-frames representing shots and identify scene boundaries [36]. Haroon et al. used scale-invariant feature transformation (SIFT) [37] to represent the shot key-frame and then identified scene boundaries comparing the transformed descriptors [38]. Sidiropoulos et al. introduced a graph-based clustering method called scene transition graph (STG) [16]. This approach connects shots together based on a selected distance measure and identifies scene boundaries by analyzing shots that lack links within a specified number of shots.

Pei et al. considered scene boundary detection a graph-linking problem in which a graph convolutional network (GCN) was trained to link shots based on the pivot graph generated using cosine similarity among shots [18]. Trojahn et al. used a video key-frames selector (VKFrameS${}^{2})$ [39] to determine the key-frames within the shots based on visual, audio, and textual features [12]. Selected key-frames were analyzed as a sequence going through an LSTM network for detecting scene boundaries. Son et al. proposed a method for fusing visual and audio features representing relational information among shots and detecting scene boundaries using a recurrent neural network (RNN) based on the fused features [21]. Baraldi et al. proposed the deep Siamese network, which evaluates the similarity among shots based on video transcript embeddings extracted from word2vec and visual features [13]. Baraldi et al. proposed an improved clustering method by minimizing the total within-group sum of squares (TWSS) of the fused and encoded shot representations based on transcript embeddings, visual frame features, and audio [11]. Protasov et al. grouped the shots based on their similarity of visual features and declared scene boundaries when the dissimilarity between clusters was more significant than a threshold [17]. Bouyahi et al. represented video segments as a four-dimensional genre vector combining visual and audio features. Scene boundaries are identified by applying an unsupervised bi-clustering technique on the representative vector [19]. Rotman et al. proposed an optimal sequential grouping (OSG) that clusters the shots by minimizing the intra-scene distance of fused visual features and audio features extracted from VGGish [14,20]. Having the number of scenes, OSG declares shot boundaries using dynamic programming.

The introduction of the transformer architecture by Vaswani et al. in their seminal paper [40] revolutionized the field of natural language processing, particularly in language translation and modeling tasks. The transformer’s success in capturing long-range dependencies and handling sequential data led to its application in various sequence modeling problems, including video segmentation. Researchers recognized the transformer’s potential in tackling sequence modeling challenges and began incorporating the full transformer architecture or attention layers into their solutions. Liu et al. proposed an adaptive context reading network (ACRNet) model based on the transformer, which encodes visual features selecting each shot as the query and the other shots in a specific range as a key-value pair [15]. Using Q-function [41], the number of scenes is estimated, and scene boundaries are detected using the normalized cuts (NCuts) [42]. Islam et al. proposed the TranS4mer model for movie scene detection, which combines self-attention, capturing short-range intra-shot dependencies, and state-space operation, aggregating the long-range inter-shot cues [43].

## 3. Method

**Figure 2:**
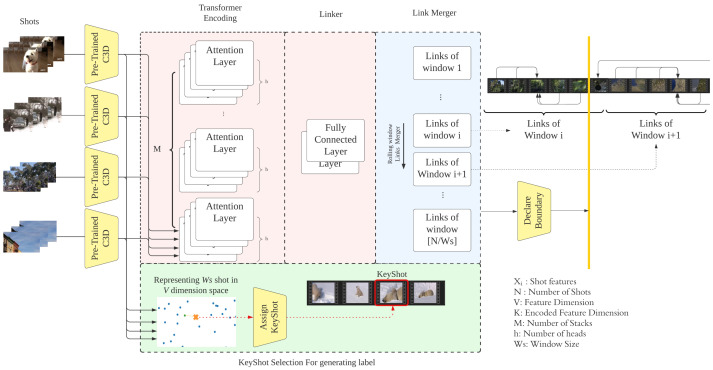
Details of proposed TELNet model.

As shown in Figure 2, TELNet identifies video scene boundaries through four stages: representing shots using pre-trained 3D CNN models, encoding shot representations, linking related shots within a rolling window, and merging the established links throughout the video. In the following sections, we provide details of every stage.

### 3.1. Shot Representation

The authors utilized an updated version of the C3D model [30], which was pre-trained on the Sports-1M dataset [44] for representing video shots. Each video shot was sampled into 16 frames, fulfilling the requirement of the C3D model, and represented by features extracted from the last layer of C3D, removing the softmax layer.

### 3.2. Transformer Encoding

**Figure 3:**
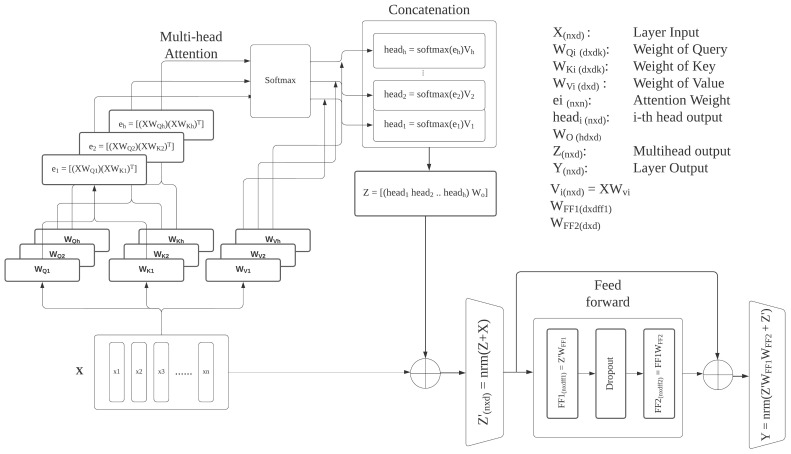
Transformer encoding layer.

TELNet relies on a combination of multi-head stacked self-attention networks as the feature encoder. Considering a video with *N* shots and with each shot represented by *d* features, *X* is denoted as the shots feature matrix where *X* ∈$R^{N\times d}$. Each attention head transforms input features using $W_{Qi}$∈$R^{d\times dk}$, $W_{Ki}$∈$R^{d\times dk}$, and $W_{Vi}$∈$R^{d\times dk}$, respectively. The attention weight for the i-th head $e_{i}$∈$R^{n\times n}$ can be written as (1). Each attention head output, denoted as $head_{i}$, is the softmax of $e_{i}$ multiplied by $XW_{v}$ (2). The multi-head output *Z* is the weighted sum of the concatenated outputs of the attention heads (3). The normalized residual output $Z^{\prime }$ is the multi-head output *Z* added to the original features *X*. $Z^{\prime }$ is then passed through a feed-forward network with weight $W_{FF1}$ and $W_{FF2}$. Each stack of multi-head attention layers’ outputs, denoted as *Y*, is the normalized feed-forward output plus $Z^{\prime }$ (4). Figure 3 illustrates the detailed operation of a single transformer encoding layer.

(1) $$e_{i}=\left[\left(XW_{Qi}\right){\left(XW_{Ki}\right)}^{T}\right]$$

(2) $$head_{i}=softmax\left(e_{i}\right)V_{i},whereV_{i}=XW_{Vi}$$

(3) $$Z=concat\left(\left[head_{1}head_{2}head_{3}\ldots head_{h}\right]\right)W_{O}$$

(4) $$Y=norm(Z^{\prime }W_{FF1}W_{FF2}+Z^{\prime })$$

Consider the multi-head attention network as a function that takes shot features *X* and generates *Y* as an output; we can run this function on itself multiple times through stacking. The output of the stacked multi-head attention network is denoted by *F*.

### 3.3. Linker

With the encoded shot features *F*, linker establishes links among shots and generates a video graph in which all shots (nodes) are linked through weighted edges. The linker estimates the edge weights using two fully connected layers, denoted by the *L* function. The *L* function takes $S_{j}$, the source shot encoded features located in the j-th row of *F*, model parameters θ, and predicts the pairwise linking probability $Y_{j}=L\left(S_{j},F,\theta \right)$, denoting the linking probability of the j-th shot to other shots in *F* (edge weight).

### 3.4. Merging Algorithm

**Figure 4:**
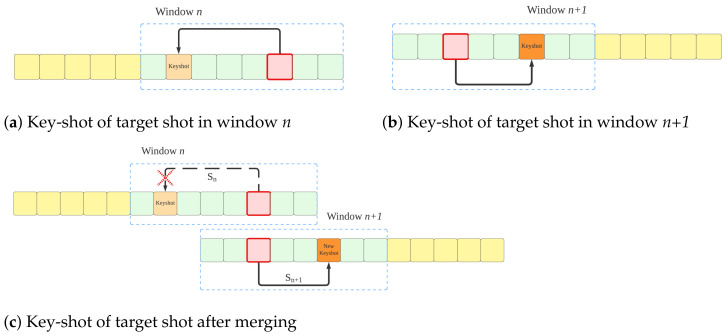
Merge algorithm. Two separate key-shot candidates of the target shot in window *n* and window *n* + 1. Compare key-shot in window *n* with key-shot in window *n* + 1, keep the most related one as the final key-shot.

TELNet analyzes a fixed number of video shots using a rolling window, and the linker establishes links among video shots within the rolling window. Through the link merging, the established links among overlapping rolling windows are compared and merged; the link with the highest edge weight will be preserved and used for detecting scene boundaries (see Figure 4). An ablation study and statistical analysis led to selecting a rolling window size of fifteen shots and a step change of ten shots in implementing TELNet.

## 4. Experiment

**Figure 5:**
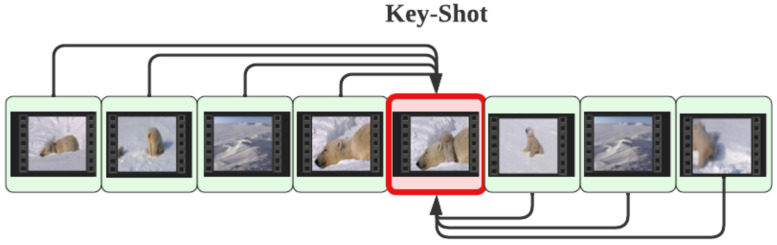
Training label generation. This diagram represents a sample scene in which all the other shots within a scene are linked to the key-shot (red rectangle).

TELNet generates a video graph in which shots (nodes) are linked based on their relationships, quantified by edge weights. We transform raw shot features into encoded representations through the training process, wherein intra-scene shot features are brought closer together than out-of-scene shot features. In simpler terms, the encoded features capture the semantic relationships among intra-scene shots learned from the ground truth label. Having access to labeled datasets that provide scene boundaries, we have devised the graph label generation procedure, which involves two steps:

1. Selecting the key-shot as the closest shot to the mean of the shot features within a scene (refer to Algorithm 1). In contrast to the maximum variance method, which aims to select the most diverse shot within a scene, we have proposed the key-shot selection based on its proximity to the mean of the shot features. The rationale behind our approach is to identify a shot that can effectively represent the storyline of the scene, encapsulating the most common visual content. By choosing the key-shot as the one closest to the mean, we prioritize shots that align with the overall visual theme of the scene, leading to a more cohesive and representative graph. While the maximum variance method may emphasize shots with diverse characteristics, it may include outliers that do not accurately depict the primary content of the scene. In contrast, our proposed method seeks to find a shot that best encapsulates the central theme, ensuring that the key-shot serves as a reliable anchor for connecting other intra-scene shots. This approach enhances the interpretability and coherence of the video graph, enabling more accurate analysis and understanding of the video content.
2. Establishing links among intra-scene shots to the key-shot (refer to Figure 5). This step involves connecting the key-shot and other shots within the same scene. The final graph effectively captures the cohesive relationships among the shots, providing valuable insights into the scene’s content.

The transformer encoder and the linker were trained together, minimizing the cross-entropy loss in the predicted linking probabilities $Y_{j}=L\left(S_{j},F,\theta \right)$ and the ground truth graph label generated $Y_{j-Label}$:

(5) $$Loss=-\sum_{j=1}^{N}Y_{j-Label}log\left(L\left(S_{j},F,\theta \right)\right)$$

**Algorithm 1** Key-shot selection, graph label generation**Input**: Raw shot features within a scene**Output**: *Y*${}_{j-Label}$, label
  1:Consider j-th scene containing n shots starting from *k*-th shot in a video  2:Mean shot feature $f_{mean}=\frac{1}{n}\sum_{i=k}^{k+n-1}f_{i}$  3:minDist = *∞*  4:**for***i* = *k* to *k* + n − 1 **do**  5:    Dist = EuclideanDistance ($f_{mean},f_{i}$)  6:    **if** Dist < minDist **then**  7:        minDist = Dist.  8:        Key-Shot = *i* # Index of Key-shot  9:    **end if**10:**end for**11:*Y*${}_{j-Label}$ = $\left[\mathrm{Key}-\mathrm{Shot}\right]\times n$12:**return***Y*${}_{j-Label}$

### 4.1. Implementation Detail

Shot features extracted from the pre-trained 3D CNN $X\in R^{N\times 4096}$ are transformed to encoded features $F\in R^{N\times 4096}$ using a four-head, six stacks of self-attention networks—N is the number of shots in a video. The linker is implemented using a three-layer fully connected neural network, as detailed in Table 2.

The proposed model consists of 514 million parameters. All experiments were conducted using PyTorch version 1.9.0 with Cuda version 11.4. The training and evaluation processes were carried out on a server equipped with an Intel i7-10700K processor, 64 GB of memory, and an Nvidia RTX A5000 24 GB graphics card.

### 4.2. Datasets

The following datasets were used to train and evaluate the proposed model.

#### 4.2.1. BBC Planet Earth Dataset

The BBC Planet Earth dataset comprises approximately 9 h of videos, containing 11 episodes of the BBC Planet Earth program. Each video is labeled with shot and scene boundaries, with an average of 8.5 shots per scene [13]. The specifics of the video contents in the BBC Planet Earth dataset are presented in Table 3.

#### 4.2.2. OVSD Dataset

The Open Video Scene Detection (OVSD) dataset, proposed by Rotman et al., comprises 21 movies and animations [20]. However, due to copyright issues at the time of writing the paper, only nine videos, totaling approximately 12 h of content, were accessible for use. In the OVSD dataset, scene boundaries are already marked. To identify shot boundaries, the authors utilized the KTS (kernel temporal segmentation) algorithm [24], resulting in an average of 12.6 shots per scene during their analysis. The specifics of the video contents in the OVSD dataset are presented in Table 4.

#### 4.2.3. MSC Dataset

The Movie SceneClip (MSC) dataset, introduced by Liu et al., consists of 500+ videos [15]. Unlike the BBC Planet Earth or OVSD datasets, the MSC dataset contains movie video highlights rather than complete videos. Following previous studies, the authors combined videos from the same movie to create videos with pseudo-scene boundaries, resulting in an average of 34 shots per scene for 468 videos. Video clips can be downloaded from the Fandango MovieClips Youtube channel [45]. Table 5 shows the video detail of MSC dataset used in this study.

### 4.3. Evaluation Metrics

Video scene boundary detection can be treated as a binary classification task, where the model’s performance can be assessed using average precision. TELNet achieved an average precision of 18.5 on the BBC dataset and 18.1 on the OVSD dataset. However, a limitation arises as precision and recall can only evaluate the model’s ability to precisely identify the exact scene boundaries rather than the representation of each segmentation. Due to this limitation, relying solely on average precision as the evaluation metric for scene boundary detection may lead to underestimating the model’s actual performance. An alternative evaluation metric that addresses this issue is the F-score, calculated as the harmonic mean of coverage, $C_{t}$, and overflow, $O_{t}$, which has been widely used for evaluating the performance of scene boundary detection models [46]:

(6) $$C_{t}=\frac{Z_{i=1,\ldots m}\#\left(Z_{i}\cap Z_{t}^{\prime }\right)}{\#(Z_{t}^{\prime })}$$

(7) $$O_{t}=\frac{\sum_{i=1}^{m}\#\left(Z_{i}\setminus Z_{t}^{\prime }\right)*min\left(1,\#\left(Z_{i}\cap Z_{t}^{\prime }\right)\right)}{\#\left(Z_{t-1}^{\prime }\right)+\#\left(Z_{t+1}^{\prime }\right)}$$

(8) $$F-score=\frac{2}{\frac{1}{c_{t}}+\frac{1}{1-O_{t}}}$$

The predicted scenes are denoted as $Z=\{Z_{1},Z_{2},Z_{3},\ldots ,Z_{m^{\prime }}\}$, where $Z_{i}$ represents the scene index and $i\in \{1,\ldots ,m^{\prime }\}$. The ground truth scenes are denoted as $Z^{\prime }=\left\{Z_{1}^{\prime },Z_{2}^{\prime },Z_{3}^{\prime },\ldots ,Z_{m}^{\prime }\right\}$, where $Z_{j}^{\prime }$ represents the scene index and j∈{1,…,m}. $\#(Z_{i})$ is the number of shots in the predicted *i*-th scene. TELNet achieved an F-score of 0.74 on the BBC dataset and 0.72 on the OVSD dataset.

### 4.4. Performance Comparison

Table 6 and Table 7 present a comprehensive comparison of the F-score results of TELNet against state-of-the-art (SOTA) models on the individual videos from both the BBC and OVSD datasets. The bold number indicate the highest score among different methods. Additionally, Table 8 provides a comparison of the F-score results of SOTA models on the MSC dataset. The F-score values were obtained from the original research papers or by evaluating the source code provided by the respective authors, as indicated in the tables.

To maintain consistency with prior studies, the evaluation was conducted using the leave-one-out training approach. Notably, TELNet demonstrates outstanding performance, surpassing all other models, in several cases by a significant margin. Furthermore, it achieves results on par with ACRNet, which is a more complex model [15].

Table 9 and Table 10 provide a comparative analysis of TELNet and ACRNet [15] in both canonical and transfer settings.

- In the canonical setting, the F-score results on the BBC and OVSD datasets are obtained by averaging the leave-one-out results. For the MSC dataset, the F-score is calculated by splitting the dataset into 70% for training and 30% for testing.
- On the other hand, in the transfer setting, the F-score is calculated using a full dataset for training and a different dataset for testing. In this scenario, TELNet exhibits superior performance over ACRNet, indicating the effectiveness of the proposed encoder–linker in learning the essential video structure for scene boundary detection, independent of the specific video subjects.

These findings suggest that TELNet’s architecture enables it to generalize well across different datasets, making it a robust and versatile model for video scene boundary detection tasks. The results highlight the advantages of TELNet over ACRNet, particularly in scenarios where a model’s ability to adapt to new video content is crucial.

### 4.5. Complexity Comparison

As videos become longer and contain more shots, the computational demands of scene boundary detection algorithms can escalate significantly. Therefore, it is essential to analyze and compare different models’ time and space complexities to ensure their feasibility and efficiency for real-world use.

The optical scene graph (OSG) algorithm, as described in the work of Rotman et al. [14,20], has a time complexity of $O\;(N^{2}\times M)$ when applied to a video with N shots and M scenes. Moreover, the recursive implementation of the OSG algorithm incurs a space complexity of $O\;(N^{2})$, making it more challenging to execute on personal computers due to its memory-intensive nature.

ACRNet, proposed by Liu et al. [15], utilizes the normalized cuts (Ncuts) technique to identify scene boundaries, which is an NP-hard problem [47]. Furthermore, estimating the number of shots when using ACRNet introduces additional complexities that are not considered in this comparison.

TELNet adopts a different approach. It establishes links among shots by calculating pairwise linking probabilities using a rolling window size of *Ws*. For a video with N shots, TELNet’s time complexity is $O\left(\frac{N}{Ws}\times Ws^{2}\right)$. This approach allows TELNet to identify scene boundaries even for long videos efficiently.

### 4.6. Results Sample

**Figure 6:**
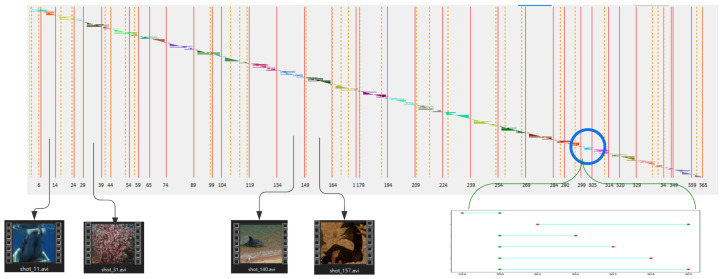
Comparison of predicted scene boundaries and ground truth labels for video 08 of the BBC Planet Earth dataset, titled “Ocean Deep”.

Figure 6 visualizes the qualitative results of applying the proposed TELNet model on the BBC Planet Earth dataset video 08, titled “Ocean Deep”.

The x-axis represents the shot indexes, the vertical orange dashed lines indicate the ground truth scene boundaries in the video, and the red vertical solid lines, on the other hand, represent the predicted scene boundaries generated by the TELNet model. The colored horizontal lines illustrate the links between shots (see the zoomed section of the plot).

If there are no horizontal lines with the same color crossing two adjacent shots, it implies the presence of a scene boundary. For instance, the absence of a light blue horizontal line crossing shot no. 149 indicates that shot no. 149 is a scene boundary.

## 5. Conclusions

This study aimed to design a simple video scene detection network that could be trained end-to-end and that relies solely on shot representations with no need for any information about video scenes, such as the number of scenes in a video.

We have proposed a model integrating a transformer encoder and a linker to identify video scene boundaries. The proposed model was trained and evaluated on multiple datasets using a novel key-shots selection technique and corresponding video graph labels. The proposed model scans shot representations extracted from the pre-trained 3D CNN model in a sequence using a rolling window methodology, encodes representations using a transformer encoder, and generates an aggregated video link graph using a linker.

TELNet achieved F-score results comparable to the other state-of-the-art models in the canonical setting and outperformed results in the transfer settings without prior knowledge of the video structure, such as the number of scenes in a video. State-of-the-art results in the transfer setting confirm that combining the selected shot representations and the transformer encoder can effectively learn the video structure of a wide range of subjects.

An equally crucial aspect of TELNet is its linear growth in computational complexity as the number of shots increases. This makes TELNet the simplest and most suitable among the state-of-the-art models for handling long videos and ideal for real-time streaming applications.

In conclusion, our study successfully introduces TELNet as an efficient and effective solution for video scene boundary detection. Its capacity to learn without prior knowledge of scene numbers and its scalability for handling longer videos highlights its applicability in various scenarios. By presenting a simple yet robust end-to-end trainable model, we aspire to pave the way for further advancements in video scene analysis.

Future directions may involve the fusion of visual, textural, and audio features to achieve context-aware segmentation. These multimodal features can enhance the model’s understanding of video content, leading to more accurate and meaningful scene boundary predictions. One promising approach is constructing a video context graph that combines visual, textural, and audio information, enabling downstream tasks such as segment scoring and video summarization. This graph-based representation facilitates ranking and scoring segments based on their relevance and importance in the overall narrative, thereby capturing the essence of video content more effectively.

## Figures and Tables

**Table 1:** Comparison of the video scene detection models, where *N* denotes the number of shots.

Model	Shot Feature	Feature Encoding	Shot Clustering	Complexity	Prior Knowledge
Sidiropoulos et al. [16]	Color Histogram + audio		Scene Transition Graph (STG)		Not required
Triplet [11]	2D CNN + MFCC + textual	DNN with triplet loss	Temporal Aware Clustering	O ($N^{3}$)	
Kishi et al. [9]	SIFT + MFCC		Off-the shelf STG, etc.		
Trojahn et al. [12]	ConvFeats + MFCC + textual		LSTM		
SDN [13]	2D CNN + textual		Siamese Network		
SAK-18 [17]			Overlapping Link		
Pei et al. [18]	2D CNN		GCN		
Bouyahi et al. [19]	2D CNN + audio		Bi-Clustering		
OSG [20]			Optimal Sequence Grouping (OSG)	O $\left(N^{2}\right)$	Number of scenes required
OSG-Triplet [14]		Triplet loss			
ACRNet [15]	3D CNN	Self-attention	Normalized Cuts (NCuts)	NP-hard	
SDRS [21]	3D CNN + audio		GRU		
TELNet	3D CNN	Transformer encoding linker		O (*N*)	Not required

**Table 2:** Transformer encoding linker setting, d model is the output dimension of transformer encoder.

Transformer Encoder	Head = 4, Number of Stacks = 6, d Model = 4096
Fully connected layer 1	(4096, 2048), activate function= ReLU
Fully connected layer 2	(2048, 1024), activate function = ReLU
Fully connected layer 3	(1024, rolling windows size)

**Table 3:** BBC Planet Earth dataset.

Video Name	Video Length	Number of Shots	Number of Scenes	FPS	Resolution
From Pole to Pole (01)	49:15	445	46	25	360 × 288
Mountains (02)	48:05	383	44		
Ice Worlds (03)	49:17	421	48		
Great Plains (04)	49:03	472	57		
Jungles (05)	49:14	460	54		
Seasonal Forests (06)	49:19	526	52		
Fresh Water (07)	49:17	531	57		
Ocean Deep (08)	49:14	410	46		
Shallow Seas (09)	49:14	366	58		
Caves (10)	48:55	374	53		
Total	8:59:53	4855	568		

Source: https://aimagelab.ing.unimore.it/imagelab/page.asp?IdPage=12 (accessed on 12 June 2023).

**Table 4:** OVSD selected videos.

Video Name	Video Length	Number of Shots	Number of Scenes	FPS	Resolution
BBB	09:56	112	15	24	1280 × 720
BWNS	01:09:46	257	36	30	524 × 360
CL	12:10	98	7	24	1920 × 804
FBW	1:16:06	686	62	30	720 × 528
Honey	1:26:49	315	20	30	480 × 216
Meridian	11:58	56	9	30	1280 × 720
LCDUP	10:23	118	10	25	1264 × 720
Route 66	1:43:25	700	55	25	640 × 432
Star Wreck	1:43:14	1055	55	25	640 × 304
Total	11:44:37	3397	269		

Source: https://research.ibm.com/haifa/projects/imt/video/Video_DataSet.shtml (accessed on 12 June 2023).

**Table 5:** Movie SceneClip (MSC) dataset.

Number of Videos	Total Shots	Average Video Length	Average Number of Shots
468	16,131	2:35	34

**Table 6:** Comparison of F-score on BBC Planet Earth dataset.

Video Name	Triplet [11]	Kishi et al. [9]	Trojahn [12]	SDN [13]	SAK-18 [17]${}^{1}$	SDRS [21]	Pei et al. [18]	Bouyahi et al. [19]	OSG [20] ${}^{1}$	OSG-Triplet [14] ${}^{1}$	ACRNet [15]	TELNet
From Pole to Pole (01)	0.72	0.65	0.63	0.56	0.5	0.78	0.57	0.48	0.66	0.68	**0.83**	0.77
Mountains (02)	0.75	0.65	0.65	0.63	0.54	0.73	0.58	0.5	0.65	0.65	**0.82**	0.68
Ice Worlds (03)	0.73	0.66	0.64	0.66	0.5	0.74	0.56	0.54	0.64	0.64	**0.77**	0.69
Great Plains (04)	0.63	0.7	0.68	0.61	0.54	0.68	0.57	0.66	0.6	0.6	0.72	**0.75**
Jungle (05)	0.62	0.67	0.63	0.55	0.51	0.66	0.55		0.56	0.55	0.7	**0.74**
Seasonal Forests (06)	0.65	0.69	0.64	0.64	0.51	0.69	0.48	0.59	0.58	0.61	0.7	**0.75**
Fresh Water (07)	0.67	0.67	0.66	0.59	0.53	0.73	0.58		0.54	0.56	0.7	**0.74**
Occean Deep (08)	0.65	0.64	0.67	0.64	0.38	0.66	0.55	0.68	0.65	0.66	0.73	**0.76**
Shallow (09)	0.74	0.69	0.64	0.64	0.55	0.67	0.56		0.57	0.56	**0.8**	0.7
Caves (10)	0.62	0.65	0.67	0.64	0.43	0.66	0.54	0.64	0.59	0.61	0.75	**0.77**
Deserts (11)	0.62	0.69	0.66	0.64	0.51	0.7	0.52	0.62	0.65	0.65	0.71	**0.77**
Average	0.67	0.67	0.65	0.62	0.5	0.7	0.55	0.53	0.61	0.62	**0.76**	0.74

${}^{1}$ The result is generated using the source code provided.

**Table 7:** Comparison of F-score on OVSD dataset.

Video Name	Trojahn et al. [12]	SDRS [21]	Pei et al. [18]	ACRNet [15]	OSG-Triplet [14]	OSG [20]	TELNet
BBB	0.57	0.75	0.65	0.74	0.81	**0.83**	0.69
BWNS	0.53	0.67	0.7		**0.75**	0.63	0.6
CL	0.64	0.69	0.78	0.61	0.49	0.62	**0.88**
FBW	0.57	0.55	0.58		**0.76**	0.57	0.66
Honey	0.6	0.67	0.73		0.73	0.58	**0.77**
Meridian	0.45		**0.86**		0.69	0.63	0.75
LCDUP	0.63	**0.83**	0.71		0.72	0.73	0.76
Route 66	0.63	0.55	0.64		**0.72**	0.54	0.64
Star Wreck	0.62		0.63		0.66	0.55	**0.71**
Average	0.58	0.68 ${}^{1}$	0.69 ${}^{1}$	**0.73** ${}^{1}$	0.7	0.63	0.72

${}^{1}$ The results obtained from this paper.

**Table 8:** Comparison of F-score on MSC dataset.

MSC Dataset	OSG [20] ${}^{1}$	OSG-Triplet [14] ${}^{1}$	ACRNet [15]	TELNet
Random 30% test	0.57	0.59	0.67	**0.69**

${}^{1}$ The result is generated using the source code provided.

**Table 9:** F-score of ACRNet [15] on cross-dataset.

Train / Test	MSC	BBC	OVSD
MSC	0.67	0.64	**0.63**
BBC	0.28	**0.76**	0.22
OVSD	0.29	0.23	**0.73**

**Table 10:** F-score of proposed TELNet on cross-dataset.

Train / Test	MSC	BBC	OVSD
MSC	**0.69**	0.62	0.6
BBC	**0.64**	0.74	**0.56**
OVSD	**0.64**	**0.64**	0.72

## Data Availability

Code are available upon request: https://github.com/JIA-BIN-CHANG/TELNET.git (accessed on 12 June 2023).
